# Mouthfeel of Food and Beverages: A Comprehensive Review of Physiology, Biochemistry, and Key Sensory Compounds

**DOI:** 10.1111/1541-4337.70223

**Published:** 2025-07-08

**Authors:** Katarzyna Wolinska‐Kennard, Christina Schönberger, Adam Fenton, Aylin W. Sahin

**Affiliations:** ^1^ BarthHaas UK Paddock Wood UK; ^2^ School of Food and Nutritional Sciences University College Cork Cork Ireland; ^3^ BarthHaas GmbH & Co. KG Nuremberg Germany

**Keywords:** beer, chemosensory system, consumer sensory preferences, flavor, food and beverage, mouthfeel, oral processing, sensory compounds, somatosensory system, texture

## Abstract

Mouthfeel is a complex and multidimensional sensory experience that plays an important role in how people perceive flavor and accept food and beverages. Although it is a key part of the eating and drinking experience, mouthfeel is still not clearly defined and is difficult to measure. This review explores mouthfeel from a flavor perspective by examining the physiological processes, chemical interactions, and main sensory qualities involved, such as viscosity, crunchiness, astringency, and thermal sensations like cooling and warming. During consumption, food and beverages release a variety of chemical components in the mouth. These are broadly classified as volatile and non‐volatile compounds. Volatile compounds, which act as odorants and some irritants, stimulate the olfactory system and contribute to aroma perception. In contrast, non‐volatile compounds include tastants, irritants, and texture‐related molecules that activate the gustatory and trigeminal systems. The integration of these chemical and physical signals from multiple sensory pathways is essential to flavor perception and underpins the experience of mouthfeel. The roles of oral processing, saliva, and the sensory systems, particularly the trigeminal system, are discussed to better understand how mouthfeel is created and perceived. The review also looks at current sensory and instrumental methods used to evaluate mouthfeel, outlining their uses and limitations. Beer, especially non‐ and low‐alcoholic beers, is used as a case study to show how mouthfeel affects product development and consumer acceptance. By combining insights from sensory science, food chemistry, and neuroscience, this review highlights the need for better tools and clearer frameworks to study and improve mouthfeel in modern food and beverage products.

## 1 Introduction

Flavor is a key factor in consumer satisfaction and food acceptance, and it is shaped by the combined input of multiple senses: taste, smell, and mouthfeel (Agorastos 2023; International Organization for Standardization 2008; Jean‐Xavier Guinard 1996; Spence et al. 2014).

Although taste and aroma have been widely studied, mouthfeel remains less understood despite its significant role in the overall sensory experience (Ditschun et al. 2025; Fox et al. 2022; Mouritsen and Styrbaek 2017; Selway and Stokes 2013).

Mouthfeel or mouth‐feel (S. Q. Liu 2015) is a complex sensory perception that involves the physical, tactile, and textural sensations experienced in the mouth when consuming food or beverages (Fox et al. 2022). Research on texture began in the late 19th and early 20th centuries (Bourne 1982; Szczesniak 2002), but it was not until the late 1950s that texture started to be studied as its own topic, similar to how flavor had been explored earlier (Szczesniak 2002). Mouthfeel is more than a singular attribute (Ditschun et al. 2025), and although it plays a significant role in shaping consumer preferences (Chong et al. 2020; Jean‐Xavier Guinard 1996; Linne et al. 2023), its definition remains ambiguous and open to interpretation. It is also often used interchangeably with texture, despite the two terms referring to different sensory modalities (Selway and Stokes 2013; Weenen et al. 2005, 2003). Texture as mouthfeel is multidimensional (Jean‐Xavier Guinard 1996; Stokes et al. 2013); however, it describes the mechanical and structural properties of food such as firmness, crunchiness, or viscosity and can often be measured instrumentally (Jean‐Xavier Guinard 1996; Kohyama 2020). By comparison, mouthfeel lacks well‐established techniques and instruments for accurate measurement (Ditschun et al. 2025). Although the relationship between mouthfeel and texture is not clearly defined, both play a critical role in product acceptance and sensory experience (Chong et al. 2020; Stokes et al. 2013).

Mouthfeel is commonly evaluated using sensory panels (H. Lawless and Heymann 2010), though these methods are expensive and resource‐intensive (Fox et al. 2021). Instrumental approaches, such as rheology, soft tribology (Fox et al. 2021; Shewan et al. 2020), electronic tongues (Bleibaum et al. 2002; Polshin et al. 2010), and QCM (quartz‐crystal microbalance) sensors (Kaneda et al. 2002, 2003, 2005), offer faster and more objective alternatives, particularly suited for texture analysis. However, there is still a lack of reliable instrumental techniques specifically tailored to fully evaluate mouthfeel (Ditschun et al. 2025).

Mouthfeel is different from taste, which involves the interaction of food's chemical compounds with taste receptors (Fox et al. 2021) or smell, the sensory perception of volatile chemical compounds through olfactory receptors in the nasal cavity (H. Lawless and Heymann 2010). Mouthfeel relates to the physical sensations triggered by the food's texture, viscosity, and other mechanical properties, detected by chemosensory and somatosensory receptors in the oral cavity, primarily transmitted by the trigeminal nerve (van Eck et al. 2019). The sensory attributes that influence mouthfeel are caused by multiple compound classes, which suggests the involvement of diverse physiological mechanisms (Gawel et al. 2000; Jean‐Xavier Guinard 1996). Texture can be described as the sensory and functional expression of a food's structural, mechanical, and surface properties, as perceived through the senses of sight, sound, touch, and movement (kinesthetics) (Marciniak‐Lukasiak et al. 2024; Szczesniak 2002).

Additionally, the role of saliva is critical in flavor perception and mouthfeel, as it acts as a medium for dissolving flavor compounds and modulating textural sensations. Its composition and flow influence how attributes like smoothness, astringency, and coating are perceived, making it an essential factor in the overall sensory experience (Agorastos, van Halsema, et al. 2023; Canon et al. 2018; Muñoz‐González, Feron, et al. 2018; Selway and Stokes 2013).

The importance of mouthfeel lies in its influence on overall flavor perception and is an important indicator of acceptance and liking by consumers (Fox et al. 2022; Guinard and Mazzucchelli 1996; Stokes et al. 2013). In some products, the texture is found to be more significant than the flavor itself (Kumar and Chambers 2019). The perception of food and beverages is influenced by every aspect of mouthfeel, including smoothness and grittiness (Gupta et al. 2022). For example, a creamy yogurt with a smooth texture is often perceived as fresher and of higher quality than a gritty one, influencing consumer choices (Gupta et al. 2022). Chocolate's texture, including its particle size, particle distribution, and ingredient composition, affects how it melts and feels in the mouth, shaping its flow and sensory characteristics (De Pelsmaeker et al. 2019; Malvern Panalytical 2016; Toker et al. 2023). Exploring the rheological properties of chocolate, a non‐Newtonian substance, offers valuable insights for food scientists to improve and optimize product quality and manufacturing processes (Toker et al. 2023). Conversely, undesirable texture can lead to food rejection and is a key driver of food aversion (A. Drewnowski 1997; Jeltema et al. 2016).

Despite its significance, mouthfeel is frequently disregarded and overlooked in the overall flavor experience (Mouritsen and Styrbaek 2017). The mechanical aspects of eating and drinking that produce tactile sensations underlying mouthfeel are rarely considered (Mouritsen and Styrbaek 2017). Understanding mouthfeel is essential as it directly influences consumer preferences and the acceptability of food products (Fox et al. 2022; Guinard and Mazzucchelli 1996). Characteristics, such as creaminess, crunchiness, and lightness (bubbly sensation), or even multi‐layered mouthfeel, have a big influence on people's perception and enjoyment of food and beverages, and they also affect success in the market (Clark 1998). Furthermore, the interaction between taste and mouthfeel influences the overall flavor experience (Mouritsen and Styrbaek 2017). Tastants are chemical compounds that stimulate taste receptors and interact with taste buds, triggering sensations like sweetness, bitterness, sourness, saltiness, and umami. These compounds can alter mouthfeel by changing food's rheological properties (D. Liu et al. 2017). For instance, sugars enhance viscosity, body, smoothness perception, and overall mouthfeel (Bajwa and Mittal 2015; D. Liu et al. 2017), whereas acids increase astringency and dryness (D. Liu et al. 2017). This interaction underscores mouthfeel's complexity and integral role in the sensory experience and key compound of flavor.

The aim of this review, while primarily focused on mouthfeel and texture in food and beverages, is to highlight non‐ and low‐alcoholic beer as a unique sensory and formulation challenge in meeting evolving consumer expectations. This serves as a clear example of how the mouthfeel of beer, historically overlooked, can have a significant impact on overall product acceptability and consumer liking. The final section is dedicated specifically to beer, exploring its sensory characteristics, mouthfeel contributors, and shifting market demands. Beer is not only one of the oldest alcoholic beverages (Buiatti 2009; Raihofer et al. 2022) but also among the most widely consumed worldwide, ranking third after water and tea (Agorastos, Klosse, et al. 2023; Piazzon et al. 2010). As the craft and non‐alcoholic beer segments continue to grow (Bellut and Arendt 2019; Fox et al. 2022). Sensory attributes beyond flavor and aroma, particularly mouthfeel, have gained increasing importance in consumer acceptance and product development (Gabrielyan et al. 2014; Krebs et al. 2019). Mouthfeel of beer encompasses sensory properties such as carbonation, smoothness, fullness, astringency, and mouth‐coating and is integral to the overall beer drinking experience (Agorastos, Klosse, et al. 2023; Ivanova et al. 2023; P. Klosse 2013). Although beer contains hundreds of chemical compounds, the specific contributions of individual molecules to mouthfeel remain underexplored (Agorastos, Klosse, et al. 2023). Recent advances in sensory science and frameworks, such as Klosse's three‐dimensional model, coating, drying, and contracting, offer promising tools to predict and classify mouthfeel attributes based on chemical composition (Agorastos, Klosse, et al. 2023; P. Klosse 2013), supporting brewers in designing more balanced and appealing products.

## 2 Understanding Flavor Terminology

### 2.1 The Language of Flavor

To fully appreciate mouthfeel, it is essential to understand its relationship with other sensory aspects, including taste, taste perception, smell, and taste sensitivity (how well or intensely a person perceives different tastes and smells), as well as threshold (the minimum concentration of a substance required for detection), tasting, taste percept, and attributes that are specific for the product. During consumption, food and beverages release a range of chemical components into the mouth that are broadly classified as volatiles and non‐volatiles.

Volatile compounds are small molecules with low molecular weight (LMW) and high vapor pressure, enabling them to evaporate easily at room or body temperature. They are defined as substances capable of evaporating under normal conditions and are typically responsible for odor and aroma perception via the olfactory system (International Organization for Standardization 2008; Zarzo 2007). Once transported to the nasal cavity, these compounds act as odorants and, in some cases, as irritants that activate olfactory receptors and contribute to the perception of flavor (Shepherd 2012; Small et al. 2005; Zarzo 2007).

Non‐volatile compounds are substances that do not readily evaporate under normal temperature and pressure conditions. In sensory analysis, they are typically associated with taste and mouthfeel attributes (International Organization for Standardization 2008). These include tastants, irritants, and textural compounds. Textural compounds are non‐volatile components that influence the mechanical and structural properties of food—such as firmness, viscosity, crunchiness, and elasticity, which are perceived through the somatosensory system during oral processing. These attributes contribute to mouthfeel and are detected by mechanoreceptors and proprioceptors in oral (Gawel et al. 2000; Jean‐Xavier Guinard 1996; Szczesniak 2002). Tastants are hydrophilic molecules such as sugars, salts, amino acids, and organic acids that activate taste receptors of the gustatory system (Di Lorenzo 2021). Irritants and textural elements—such as capsaicin, polyphenols, fats, and polysaccharides, are detected by the trigeminal system, contributing to sensations like astringency, viscosity, and mouth‐coating (Guinard and Mazzucchelli 1996; P. Klosse 2013).

Taste specifically refers to the detection of chemical compounds through specialized receptors located on the tongue and in the oral cavity, triggering neural responses processed in the brain to create the perception of taste (G. K. Beauchamp and Mennella 2009; Breslin 2013; Breslin and Spector 2008; Purves and Mark 2012). An important related concept is the threshold, the minimum concentration required for detection. Although taste thresholds are linked and serve as reliable indicators of general taste sensitivity (Lim et al. 2022), there is no direct correlation between taste and smell sensitivity (Lim et al. 2022; Lundstrom et al. 2012). Although taste, smell, and chemical irritation (chemesthesis) collectively shape flavor perception and interact in the brain (Green et al. 2005; Lim et al. 2022; Lundstrom et al. 2012), research suggests that taste sensitivity is largely independent of smell and chemesthetic sensitivity (Green 2004; Green et al. 2005; Lim et al. 2022; Lundstrom et al. 2012). This indicates some overlap in cortical processing, but no single factor determines overall chemical sense sensitivity (Green et al. 2005; Lundstrom et al. 2012). In contrast, mouthfeel attributes like viscosity and astringency are more complex and context‐dependent, lacking universally defined sensory thresholds. Their perception often varies based on the food matrix and individual differences, making them more challenging to quantify (Agorastos, van Halseman, et al. 2023; Norton et al. 2021; Szczesniak 2002).

Additionally, descriptors such as body, which refers to the fullness, viscosity, richness, consistency, smoothness, and compactness of a product's texture, flavor (Gawel et al. 2000; Krebs et al. 2019; Ramsey 2020; Szczesniak 1963), and palate fullness, the perceived weight, resistance to flow, and qualities related to viscosity and density (Moreno Ravelo et al. 2024), are multidimensional terms that are not easily linked to discrete sensory thresholds. Furthermore, they are often used interchangeably and inconsistently.

Understanding individual differences in taste sensitivity is also crucial for appreciating mouthfeel, as these differences shape consumer preferences (Lim et al. 2022). Sensitivity and perception may vary due to genetic variation (Diószegi et al. 2019; Williams et al. 2016), age (Lim et al. 2022), sex (Williams et al. 2016), and ethnicity, with studies showing that, for example, African Americans and Hispanics often rate taste sensations higher than non‐Hispanic Whites, especially among males (Williams et al. 2016).

Tasting, a component of oral processing, involves placing food in the mouth, where it interacts with saliva and taste receptors, initiating gustatory perception (Agorastos, van Halseman, et al. 2023; Herz 2016; Rozin 1982; Small 2012). Taste perception refers to the brain's interpretation of sensory input from taste receptors, integrating signals from taste, smell, texture, and temperature to form a complete flavor experience (Agorastos et al. 2020). The taste percept is the mental representation or conscious experience of this input, encompassing aspects like intensity, quality, hedonic value, localization, and aftertaste (Breslin and Spector 2008; Green et al. 2005).

It is also important to note that the terms “taste” and “flavor” are often used interchangeably in everyday language, though they are not synonyms (Nargi 2018; Spence et al. 2014). Additionally, taste can be confused with smell, particularly when both stimuli are present at the same time (Auvray and Spence 2008; Stevenson 1999). These sensory overlaps can lead to perceptual interactions, where aromas influence how we perceive taste, like sweet scents enhancing the perception of sweetness (Auvray and Spence 2008; Cliff and Noble 1990; Frank and By 1988; Stevenson 1999). For example, the addition of caramel aroma to a sucrose solution has been shown to enhance perceived sweetness compared to a control sample (Stevenson 1999). Similarly, the incorporation of strawberry aroma has been found to increase the perceived sweetness of whipped cream (Frank and By 1988).

Flavor is defined as the combined experience of taste, smell, and other sensations involving the olfactory, gustatory, and trigeminal systems (Agorastos 2023; Guinard and Mazzucchelli 1996) and by ISO as a complex combination of gustatory, olfactory, and trigeminal sensations perceived during tasting (International Organization for Standardization 2008). By contrast, mouthfeel is generally described as the tactile, irritant, and thermal sensations perceived in the mouth, mediated by chemosensory and somatosensory receptors (Gawel et al. 2000; Jean‐Xavier Guinard 1996). Recent studies in psychology and cognitive neuroscience further highlight the importance of multisensory interactions, demonstrating that mouthfeel, taste, and smell work together to shape our overall perception of flavor (An et al. 2023; C. Chen et al. 2024; Prescott 2015; Small et al. 2005; Spence et al. 2014). Although aroma and taste can influence the perception of mouthfeel, the specific effects can vary depending on the compound, its concentration, and the beverage and food matrix. For example, research on red wine found that aroma did not generally affect taste or mouthfeel perception, with the only notable impact being on oily mouthfeel (Ferrero‐del‐Teso et al. 2024). In contrast, another study demonstrated that sugar‐reduced drinks inevitably lead to a diminished sense of mouthfeel (Miele et al. 2017). Consumers often characterize diet or sugar‐free beverages as thin or watery, and many view these sensations negatively (Miele et al. 2017). Smell, taste, and mouthfeel are integrated components of flavor and can influence the perception of one another.

Additionally, the language and descriptors used to evaluate mouthfeel vary widely between products, with notable differences between foods and beverages; red wine is described with terms like astringency, acidity, and body (Ferrero‐del‐Teso et al. 2024; Gawel et al. 2000, 2017); chocolate is evaluated for creaminess, melting properties, stickiness, graininess, and coating (Afoakwa et al. 2008; De Pelsmaeker et al. 2019; Ditschun et al. 2025; Thamke et al. 2009); and in beer, key mouthfeel descriptors include carbonation, fullness, body, astringency, after‐feel, and foam (Fox et al. 2022; Langstaff and Lewis 1993; Langstaff et al. 1991a). These examples show how sensory language is tailored to the unique characteristics of each product.

### 2.2 Mouthfeel and Texture Models

Food scientists use various models to describe mouthfeel and texture. Klosse (2013) model categorizes mouthfeel into three dimensions, primary axes: contracting, coating, and drying (Agorastos, Klosse, et al. 2023; P. Klosse 2013). Contraction includes factors like saltiness, acidity, and irritants (such as organic acids, CO_2_, and spices), which create a tightening sensation in the mouth. The coating occurs when a residual film, often from fats like butter, cream, or oils, remains in the mouth after swallowing. Drying, distinct from both contraction and coating, refers to the absorption of saliva by dry foods or components that draw in fluids (P. Klosse 2013). The model delivers a structured framework for understanding, evaluating, and communicating the physical sensations experienced when consuming food and beverages (Agorastos 2023; Agorastos et al. 2020).

The Hutchings and Lillford (1988) model emphasizes that texture perception is a dynamic process, monitoring changes as food is broken down in the mouth. Their three‐dimensional framework is defined by degree of structure (how structured or solid the food is), degree of lubrication (moisture content and saliva interaction), and time (duration of oral processing). It maps each food's unique breakdown path during mastication. This model represents an early hypothesis linking the physical and sensory aspects of oral processing (Hutchings and Lillford 1988).

Szczesniak (1963) introduced a systematic classification of food texture based on mechanical, geometrical, and other (moisture and fat content). The model defines key mechanical parameters, such as hardness, cohesiveness, and adhesiveness, as well as secondary parameters like chewiness and gumminess, to quantify how food behaves during oral processing. This framework laid the foundation for both sensory evaluation and instrumental methods such as texture profile analysis (TPA) (Friedman et al. 1963; Szczesniak 1963).

## 3 Physiology and Biochemistry of Mouthfeel

### 3.1 Sensory Systems, Pathways, and Receptors

Mouthfeel perception is closely integrated with other sensory modalities, including taste and smell, all contributing to the overall flavor experience (Blankenship et al. 2019; Shepherd 2006). These sensory systems interact with brain networks involved in memory, emotion, and preference, helping shape eating behavior and food choices (Blankenship et al. 2019; Shepherd 2006).

Although often discussed within the broader context of flavor, mouthfeel is a distinct and essential sensory dimension, shaped by tactile, thermal, and irritant sensations in the oral cavity. Unlike taste and smell, which are driven by specific chemical stimuli, non‐volatile tastants and volatile odorants, respectively, mouthfeel arises from interactions involving texture, viscosity, temperature, and chemesthetic effects, detected via the trigeminal and somatosensory systems (Di Lorenzo 2021; Gawel et al. 2017; P. Klosse 2013). Volatile compounds primarily influence aroma via olfaction (Purves and Mark 2012; Zarzo 2007), whereas non‐volatile compounds contribute to both taste and mouthfeel, impacting properties such as astringency, thickness, and body (Agorastos 2023; Norton et al. 2021; Szczesniak 2002). Although mouthfeel interacts with smell and taste to form a complete flavor experience, it is perceptually and neurologically distinct and often more difficult to quantify due to its multidimensional and context‐dependent nature (Jean‐Xavier Guinard 1996; Spence et al. 2014; Stokes et al. 2013).

**FIGURE 1:**
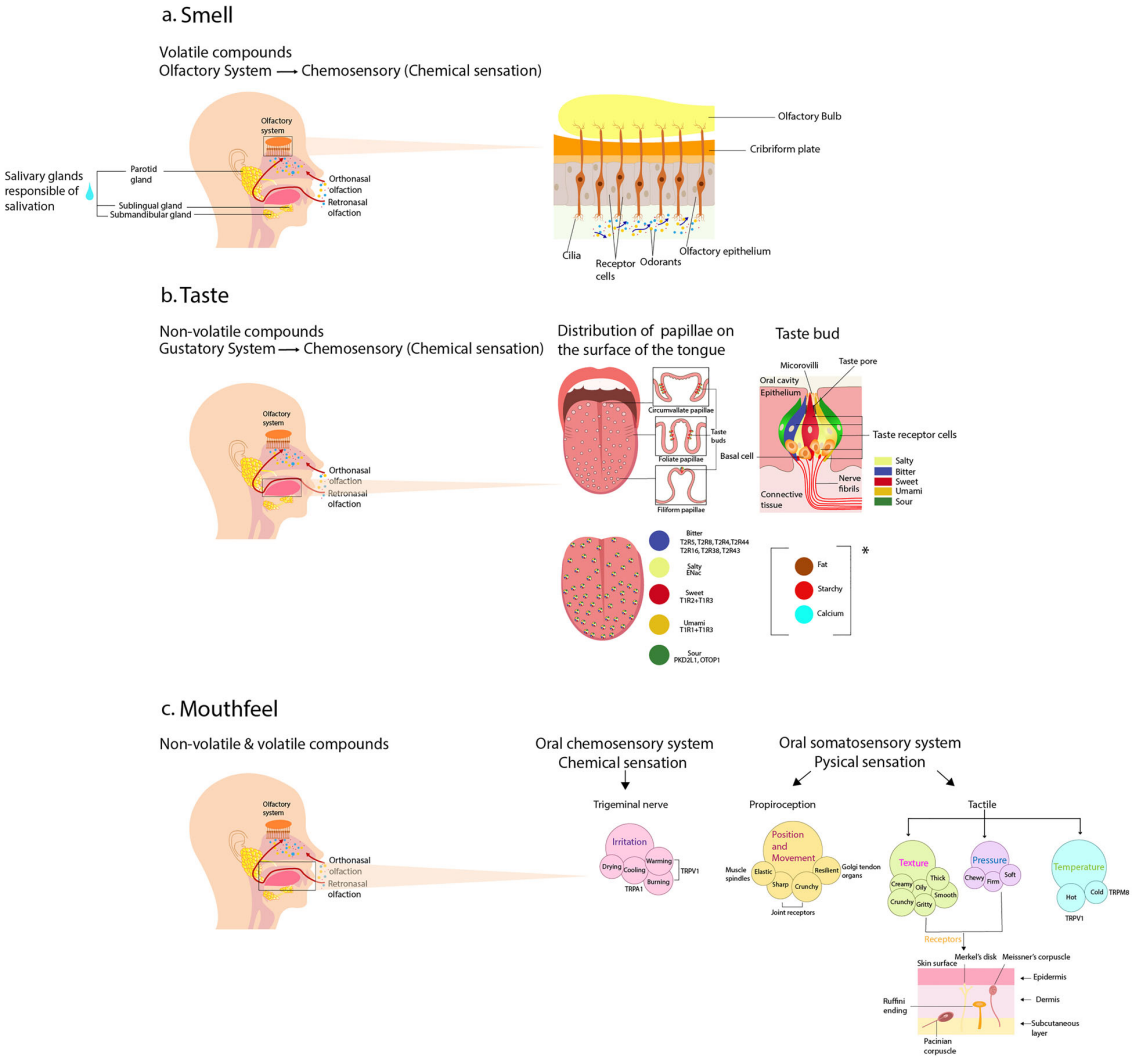
The flavor experience: integration of taste, smell, and mouthfeel sensory responses, including saliva (Adobe software): (a) **smell**, the olfactory system, including salivary glands; (b) **taste** is a second chemosensory system, also called the gustatory system. The across‐fiber model was used for the figure, TRCs within a taste bud that responds to multiple tastes (Chandrashekar et al. 2006); in the brackets potential, new tastes; (c) **mouthfeel** involves chemosensory and somatosensory systems, integrating sensations like tactile, proprioception, temperature, irritation, and pain‐related sensations (trigeminal nerve).

The olfactory system, responsible for the sense of smell, is a primary chemosensory system (Figure 1a) (Purves and Mark 2012; Spielman et al. 2019). Most researchers agree that olfaction plays a predominant role in the perception and enjoyment of food flavors (Lyman 1988; Shepherd 2012; Spence 2015). The olfactory system has several important parts and is responsible for detecting volatile compounds known as odorants (Purves and Mark 2012; Spielman et al. 2019). The olfactory epithelium (OE), located in the upper part of the nasal cavities, contains olfactory receptor neurons (ORNs) that detect odorant molecules. Axons arising from these receptor cells extend directly to neurons in the olfactory bulb (OB), which then sends projections to the piriform cortex in the temporal lobe, as well as to other structures in the forebrain via a pathway known as the olfactory tract (Purves and Mark 2012). The OBs serve as the initial area in the mammalian brain where odors are processed and represented (Weiss et al. 2020). The OB organizes these signals and then passes them on to areas of the brain, such as the piriform cortex, amygdala, and orbitofrontal cortex (Lledo et al. 2005). These brain regions further analyze the signals, allowing us to identify and recognize different smells. This step‐by‐step processing helps our brain make sense of the odors we encounter (Lledo et al. 2005). It is important to mention that humans experience smell through two main pathways: orthonasal olfaction, which occurs during sniffing through the nose from the external world, and retronasal olfaction, which happens when odorants travel from the mouth to the nasal cavities while consuming food and beverages (Blankenship et al. 2019; Small et al. 2005). Interestingly, odors can cause different brain reactions depending on how they are detected, whether they come through the nose (orthonasal) or from the back of the mouth (retronasal) (Blankenship et al. 2019; Small et al. 2005). This finding supports Rozin's idea that the way we detect odors plays an important role in how our brains interpret them (Rozin 1982; Small et al. 2005). Additionally, Blankenship et al. (2019) demonstrated that retronasal (but not orthonasal) odors share processing circuitry with taste, supporting the view that retronasal olfaction engages gustatory (taste) cortex during flavor perception. These pathways play an essential role in the perception of flavors and the overall sensory experience of food and drink (Blankenship et al. 2019; Hewson et al. 2009; Smith and Bhatnagar 2019).

**FIGURE 2:**
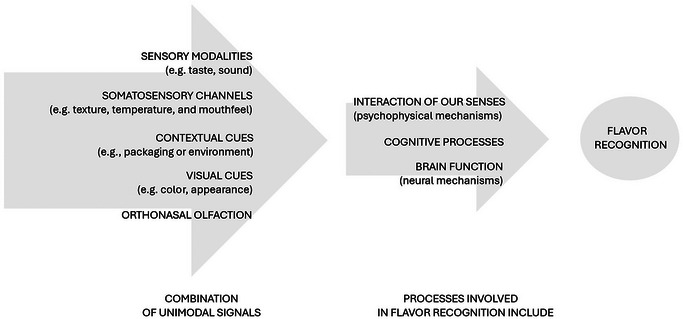
Visualization of flavor recognition: Combination of unimodal signals and processes involved in flavor recognition.

**Taste** is a second chemosensory system, also called the gustatory system (Figure 1b) (Purves and Mark 2012; Spielman et al. 2019). Chemical compounds bind to receptor proteins on taste cells, which are found in specialized epithelial structures known as taste buds on the tongue (Purves and Mark 2012). Most taste stimuli are non‐volatile compounds (Breslin and Spector 2008; Purves and Mark 2012). The concept of basic tastes is widely accepted in taste perception, typically including five tastes: sweet, salty, sour, bitter, and umami (G. K. Beauchamp 2019; Chandrashekar et al. 2006; Nelson et al. 2001). Critics argue that the term “basic tastes” lacks a clear definition or that there might be more than just these (Beauchamp 2019). In addition to the five basic tastes, there are several upcoming ones that are considered primary tastes as well, including the fat taste (Vilela et al. 2016; Jang et al. 2021; Mizuta et al. 2021), calcium (Iwata et al. 2014), starchy (glucose polymers) (Jang et al. 2021; Lapis et al. 2014), and metallic (Gonázlez Viñas et al. 1998). Nevertheless, the classification of metallic sensations as a primary taste category remains a topic of debate (Stevens et al. 2006). When discussing taste and flavor, it is essential to introduce the concepts of kokumi and koku. Kokumi is associated with the enhancement of taste perception rather than contributing a distinct taste of its own (Mizuta et al. 2021). Kokumi compounds amplify the intensity and richness of other basic taste qualities, such as sweet, salty, and umami, thereby contributing to a more complex and satisfying flavor profile (Mizuta et al. 2021). Koku is the overall sensory perception of a food's richness, depth, and mouthfulness, contributing to a more satisfying and complete flavor experience (Mizuta et al. 2021). Recent research from molecular and functional studies has shown that, in contrast to popular belief, there is no specific “map” on the tongue. All regions of the tongue are responsive to the five primary tastes (Chandrashekar et al. 2006; Nelson et al. 2001). There are two possible ways taste buds might be structured (Chandrashekar et al. 2006):

- Labeled‐line model: Each taste receptor cell (TRC) in the taste bud is dedicated to one specific taste (e.g., sweet and salty), with separate nerve fibers carrying distinct taste signals.
- Across‐fiber models: TRCs within a taste bud can either respond to multiple tastes or be specific to one taste, but nerve fibers may carry information about multiple tastes.

The TRCs are specialized for detecting different taste qualities, each mediated by specific receptors (Chandrashekar et al. 2006; Diószegi et al. 2019; Williams et al. 2016):

a.Umami:

Receptors: T1R1 + T1R3 (Kurihara and Kashiwayanagi 2000; Pal Choudhuri et al. 2016; San Gabriel and Rains 2023)

Examples of compounds causing a signal: amino acids (l‐glutamate), nucleotides (e.g., inosine monophosphate (IMP), guanosine monophosphate (GMP), adenosine monophosphate (AMP)), peptides, l‐amino acids (l‐aspartate), and derivatives of glutamate (e.g., l‐2‐amino‐4‐phosphonobutyric acid) (Kurihara and Kashiwayanagi 2000; San Gabriel and Rains 2023).

b.Sweet:

Receptors: T1R2 + T1R3 (Meyers and Brewer 2008; Sigoillot et al. 2012)

Examples of compounds causing a signal: sucrose, fructose, glucose, maltose (Meyers and Brewer 2008), artificial sweeteners (saccharin, acesulfame‐K, cyclamate, aspartame) (B. Liu et al. 2011; Simon et al. 2013; Suami et al. 1998), d‐amino acids (d‐phenylalanine, d‐alanine, d‐serine), and sweet proteins (monellin, thaumatin, curculin) (Meyers and Brewer 2008).

c.Bitter:

Receptors: T2R5 (cycloheximide), T2R8, T2R4, T2R44 (denatonium), T2R16 (salicin), T2R38 (PTC), T2R43, T2R44 (saccharin)

Examples of compounds causing a signal: quinine, strychnine, caffeine, atropine, iso‐alpha acids (hops), tetrahydro‐iso‐alpha acids, rho(dihydro)‐iso‐alpha acids, hexahydro‐iso‐alpha acids, malt‐based amino acids (e.g., l‐tyrosine, l‐tryptophan, l‐leucine, l‐threonine, and l‐phenylalanine), and tyrosol (Schoenberger and Haas Group 2006).

d.Sour:

Receptors: OTOP1 (Otopetrin 1), PKD2L1 (Polycystic Kidney Disease 2‐Like 1) (Turner and Liman 2022)

Examples of compounds causing a signal: acids (citric, tartaric, acetic, and hydrochloric) (Turner and Liman 2022), lactic acid (commonly found in sour beers), pyruvic acid, and formic acid (Bouchez and De Vuyst 2022; Dysvik et al. 2019).

e.Salty:

Receptors: Amiloride‐sensitive sodium channels, for example, ENaC (epithelial sodium channel)

Examples of compounds causing a signal: sodium chloride (NaCl) and lithium chloride (LiCl) (Anthony and Iv 2013; Bigiani 2020).

Mouthfeel (Figure 1c) involves both chemosensory and somatosensory systems, integrating sensations such as touch, proprioception, temperature, and irritation (Cayeux et al. 2023; Haggard and de Boer 2014; Viana 2011). The trigeminal nerve, often referred to as the third chemosensory pathway (Purves and Mark 2012; Spielman et al. 2019), plays a key role in this integration by detecting chemesthetic stimuli such as tingling, burning, cooling, and astringency, core elements of mouthfeel (Green 1996, 2004, 2012; Viana 2011). Although anatomically part of the somatosensory system, the trigeminal nerve also responds to chemical stimuli, contributing significantly to flavor perception (Green 1996, 2004; Lundström et al. 2011). Its broad sensory range includes touch, temperature, pain, and irritation, triggered by compounds such as capsaicin (chili), menthol (mint), and carbon dioxide (carbonated drinks). The perceived intensity of these sensations often depends on concentration levels; for instance, menthol may feel cool at low concentrations but cause burning at higher doses (Cayeux et al. 2023). These effects are mediated by transient receptor potential (TRP) channels, which play a key role in trigeminal chemoreception (Cayeux et al. 2023). Overall, the trigeminal nerve contributes to both the mechanical and chemical dimensions of mouthfeel, working in parallel with other somatosensory pathways involved in tactile feedback and oral proprioception (Green 1996; Haggard and de Boer 2014).

Oral somatosensory perception refers to the physical sensations experienced in the mouth, providing information about the structure of the oral cavity and any food present within it (Haggard and de Boer 2014). Despite its importance in shaping food preferences and contributing to mouthfeel, it remains the least understood among the major sensory systems involved in flavor perception (Lundström et al. 2011).

The oral cavity contains a rich and complex network of somatosensory receptors that contribute to mouthfeel. These include

- Mechanoreceptors, which detect touch, pressure, and vibration, are located in tissues such as the tongue, periodontal ligament, and oral (Haggard and de Boer 2014; Trulsson and Johansson 2002).
- Nociceptors, which respond to pain and temperature extremes, are primarily active in the tooth pulp and gums (Byers and Narhi 1999; Haggard and de Boer 2014).
- Thermoreceptors, responsible for detecting changes in temperature, are mainly located on the tongue (Essick et al. 2004; Haggard and de Boer 2014).

These receptors are distributed across several oral structures, each contributing to the perception of mouthfeel in specific ways (Duffy and Hayes 2020; Haggard and de Boer 2014):

- Tongue—detects texture, temperature, and pressure;
- Palate—senses texture and temperature;
- Gums—contribute to perception of firmness and structure;
- Oral mucosa—sensitive to both texture and thermal variation;
- Teeth—involved in mastication, influencing texture perception;
- Lips—detect initial contact, temperature, and surface texture as food enters the mouth.

### 3.2 Oral Processing and Saliva Interaction

Saliva plays an essential role in flavor perception by acting as a medium that dissolves and transports taste compounds to taste receptors (Agorastos, van Halsema, et al. 2023; Canon et al. 2018). However, it is surprising how rarely scientists, including cognitive psychologists and neuroscientists, mention salivation when studying taste and multisensory flavor perception (Spence 2011). It also affects the texture and mouthfeel of food, influencing how flavors are experienced. A variety of factors, such as the characteristics of food, environmental factors, and cognitive factors like attention, labeling, and mental imagery, can influence the flow rate of saliva production (Spence 2011). The proteins in saliva adhere to oral surfaces, providing a multicomponent rich in protein film (≈25 µm) (Carpenter 2012; Stokes et al. 2013). Flavor perception corresponds not quite to the characteristics of food (Canon and Neyraud 2016) mixture (Canon and Neyraud 2016). These changes in salivation can significantly impact the results of studies on multisensory flavor perception (Spence 2011) as saliva influences flavor perception, which is considered a driver of food intake (Muñoz‐González, Feron, et al. 2018). Understanding salivary dynamics is essential for accurate flavor research and practical applications in food and beverage industries (Muñoz‐González, Feron, et al. 2018). Differences in salivary parameters might have an influence on differences in perception across individuals as well as on differences within an individual throughout their life (Muñoz‐González, Feron, et al. 2018; Muñoz‐González, Vandenberghe‐Descamps, et al. 2018). For example, individuals with reduced sodium content in saliva perceived salt‐congruent aromas as greater, whereas individuals with high lipolytic activity perceived fat‐congruent aromas as more elevated (Muñoz‐González, Feron, et al. 2018).

Saliva is a multiplex mixture of fluids from the major (submandibular, sublingual, and parotid) and minor (e.g., von Ebner) salivary glands, gingival crevicular fluid, oral bacteria, cellular debris, and food debris (Muñoz‐González, Feron, et al. 2018; Neyraud et al. 2012). The salivary fluid consists of approximately 99% water, containing a variety of electrolytes (e.g., sodium, potassium) and proteins, represented by enzymes, immunoglobulins and other antimicrobial factors, mucosal glycoproteins, traces of albumin and some polypeptides, and oligopeptides (de Almeida Pdel et al. 2008).

During oral processing, food and drink components interact with saliva, a solvent for volatile and non‐volatile components, affecting oral sensations. Salivary lubrication plays a significant role in mouthfeel, influencing the integrity of the salivary film and activating mechanoreceptors (Toone et al. 2013). The viscoelasticity of food and beverages contributes to the texture characteristics of semisolid foods, impacting mouthfeel (Aktar et al. 2019).

In summary, the characteristics of saliva play a crucial role in flavor perception, including taste, aroma, and trigeminal sensations (Muñoz‐González, Feron, et al. 2018) and overall mouthfeel (Aktar et al. 2019). Moreover, saliva can influence food intake and consumption behavior (Muñoz‐González, Feron, et al. 2018, Muñoz‐González, Vandenberghe‐Descamps, et al. 2018). Understanding these factors is essential for exploring the sensory and behavioral dimensions of flavor perception.

### 3.3 Examples of Mouthfeel Attributes and Key Influencing Compounds

These mouthfeel sensations are influenced by various factors, including the chemical composition of the food or beverage (matrix), the concentration of specific compounds, and the interaction of these compounds within the food matrix. The constituents themselves can directly influence mouthfeel; however, their effects are modulated by other factors (Linne et al. 2023). A study by Ivanova et al. (2022) confirmed the multidimensional nature of body perception in both beer and wine. Rather than being a singular measure of viscosity, body was understood as a complex interplay of sensory modalities primarily driven by flavor (Ivanova et al. 2022). Although texture contributed to body perception in both beverages, it was more influential in wine. Ethanol also showed a stronger association with body in wine than in beer. Visual and aromatic cues were found to shape consumer expectations but did not directly influence the sensory perception of body (Ivanova et al. 2022). Building on these, Ivanova et al. (2023) demonstrated that perceived body in beer was positively associated with sensory attributes such as smoothness, overall flavor, overall aftertaste, and hoppy aroma, while showing a strong negative correlation with watery mouthfeel, based on Rate‐All‐That‐Apply (RATA) data (Ivanova et al. 2023). A four‐way ANOVA further revealed that ethanol, viscosity, bitterness, and aroma each significantly and positively influenced body intensity ratings, with ethanol exerting the strongest effect. The highest body scores were observed in beers where all four factors were modified simultaneously. Interestingly, bitterness had a dual effect: Although it enhanced body perception under certain ethanol levels, it also reduced overall liking at higher intensities, highlighting the importance of balanced formulation (Ivanova et al. 2023).

Table 1 provides a list of mouthfeel examples of attributes and the compounds responsible for each sensation, including the foods, beverages, and products in which they are present.

**TABLE 1:** Examples of mouthfeel attributes and key influencing compounds.

Mouthfeel attribute	Key compounds	Products	References
Creaminess	Fatty acids, triglycerides, saturated fats, proteins [non‐volatile]	Dairy products (e.g., butter and cream), chocolate	J. Chen and Stokes (2012), Drewnowski and Almiron‐Roig (2010), Szczesniak (2002), van Eck et al. (2019)
Grittiness/Graininess	Particulate matter [non‐volatile]	Ground spices (e.g., pepper), seeds (e.g., strawberry seeds)	J. Chen and Stokes (2012)
Smoothness	Polysaccharides (xanthan gum), pectin, gelatine, mono‐ and diglycerides [non‐volatile]	Gels, sauces, custards, processed foods, oils, ice cream	J. Chen and Stokes (2012), Drewnowski and Almiron‐Roig (2010), Mostafavi (2019), van Eck et al. (2019)
Astringency	Tannic acid, epigallocatechin gallate (EGCG) [non‐volatile]	Tea, wine, unripe fruits, coffee, cocoa	Charlton et al. (2002), J. Chen and Stokes (2012), Deng et al. (2022)
Drying/Mouth‐drying	Tannins [non‐volatile]	Tea, wine, coffee	Ramos‐Pineda et al. (2018)
Richness	Saturated fats [non‐volatile]	Meat, dairy products	Drewnowski and Almiron‐Roig (2010)
Crispiness	Starch, cellulose, crystalline fat, water [non‐volatile]	Potato chips, crackers, bread, fried foods, snacks, Maillard reaction products	Drewnowski and Almiron‐Roig (2010), Mollakhalili‐meybodi et al. (2023), Szczesniak (2002), van Eck et al. (2019)
Melting quality	Cocoa butter, milk fat [non‐volatile]	Chocolate, cheese	Drewnowski and Almiron‐Roig (2010)
Carbonation bite	Carbonic acid (H_2_CO_3_) [non‐volatile]	All carbonated beverages	Hewson et al. (2009)
Tingling	Carbon dioxide [volatile]	All carbonated beverages	Hewson et al. (2009)
Firmness/Tenderness	Proteins, fibers [non‐volatile]	Cheese, meat, fruit, vegetables	Szczesniak (2002), van Eck et al. (2019)
Chewiness	Protein (collagen), protein (gluten), gelatine [non‐volatile]	Meat, bread, pizza, gummy candies	Mollakhalili‐meybodi et al. (2023), Szczesniak (2002)
Toughness	Protein (collagen), fibers (cellulose), [non‐volatile]	Meat, gummy candies, low‐fat ice‐cream, salad dressings	Szczesniak (2002)
Viscosity	Polysaccharides [non‐volatile]	Sauces, soups	Szczesniak (2002)
Burning sensation	Capsaicin [non‐volatile]	Jalapeños, cayenne, and paprika	Fattori et al. (2016)
Warming sensation	Gingerol [non‐volatile], ethanol, [volatile]	Fresh ginger, ginger beer, and ginger ale, beer, wine, distilled spirits	Ickes and Cadwallader (2017), Unuofin et al. (2021)
Cooling sensation	Menthol, eucalyptol [volatile]	Peppermint, spearmint, eucalyptus oil, bay leaves, tea tree	Eccles (2011), Thiel and Rössler (2024)
Moistness	Fats, sugars [non‐volatile]	Breads, sweet doughs	Mollakhalili‐meybodi et al. (2023)
Softness	Starches, fats [non‐volatile]	Sandwich bread, brioche	Mollakhalili‐meybodi et al. (2023)
Mouth‐coating	Chlorogenic acids (3‐ and 4‐caffeoylquinic acid), polysaccharides, flavonol glycosides (e.g., quercetin 3‐glucoside), fats (saturated and unsaturated) [non‐volatile]	Coffee potatoes, rice, wheat, corn, black tea, red wine, ice cream	Linne et al. (2023), Mostafavi (2019), Ramos‐Pineda et al. (2018), Yadav and Karthikeyan (2019)

## 4 Sensory Expectation and Its Influence on Flavor

The flavor is a multisensory experience (Agorastos 2023; Drew et al. 2023; International Organization for Standardization 2008; Jean‐Xavier Guinard 1996; Pereira and van der Bilt 2016; Spence et al. 2014) that involves the integration of several senses working together (Drew et al. 2023). The flavor perception comes from the combination of unimodal signals (Drew et al. 2023; Pereira and van der Bilt 2016), including (a) sensory modalities, such as taste, smell, and sound; (b) somatosensory channels, which encompass sensory inputs related to touch and feel, such as texture, temperature, and mouthfeel, all of which are important in flavor perception; and (c) other cues, such as visual cues (e.g., color and appearance), orthonasal olfaction (smell through the nose), and contextual cues (e.g., packaging or environment) (Figure 2) (Drew et al. 2023). Furthermore, the processes involved in flavor recognition include (a) interaction of our senses (psychophysical mechanisms), (b) cognitive processes (how we think and process information), and (c) brain function (neural mechanisms) (Figure 2) (Drew et al. 2023). However, a comprehensive understanding of how these processes manage congruent or conflicting sensory information is still lacking (Drew et al. 2023).

One unique attribute of flavor in comparison to other types of perception, for example, vision or object recognition, is how closely it is connected to pleasure or enjoyment (Drew et al. 2023). Flavor encompasses both the sensory experience, which includes the specific attributes detected (known as sensory‐discriminative aspects), and the emotional response, which reflects the degree of like or dislike for the food or drink (Drew et al. 2023). However, despite much research on how senses work together, not all senses are studied equally in depth. It is recognized that our understanding of flavor processing is considerably less advanced than that of other senses, such as sight or hearing (Drew et al. 2023). Understanding customer expectations is vital for product success, as the selection of food products is affected by a combination of food characteristics, consumer motivations, emotions, and context (Kumar and Chambers 2019). The product should also provide satisfaction, indulgence, or refreshment while aligning with current health trends. Balancing the taste and feel of the product with its nutritional benefits is essential in the formulation of plant‐based alternatives (Boeck et al. 2022; Halm et al. 2024). Finally, how a product affects emotions and thoughts (psychophysiological effects) plays a role in how customers perceive and respond to it, making this an important consideration for product development (Labbe et al. 2006).

Genetic, social, and cultural aspects appear to play a key role in taste sensitivity and food preference (Birch 1999; Kurshed et al. 2022; Pereira and van der Bilt 2016). Variations in oral physiology that exist among individuals may lead to differences in food perception (Pereira and van der Bilt 2016). Additionally, the way humans perceive food and beverages is significantly influenced by our expectations and prior experiences (Aaron et al. 1994; Birch 1999). Environmental factors during eating, such as watching electronic media and TV and exposure to social media, may impact the food perception and the amount of food intake (Filippone et al. 2022; Garg et al. 2025; Pereira and van der Bilt 2016). The research on full‐fat (FF) and reduced‐fat (RF) products revealed that label information can influence consumers’ sensory and hedonic ratings; however, the impact varies based on individual attitudes and beliefs regarding these products (Aaron et al. 1994). Consumers with more positive attitudes gave higher ratings when the labels aligned with their preferences (Aaron et al. 1994), and expectations can greatly impact how consumers perceive both flavor and mouthfeel (Siret and Issanchou 2000).

The way humans experience food comes from the combination of many different sensory cues that come from a wide range of sources, and the mechanisms by which they affect the flavor experience are diverse. These cues can be categorized into two types: interoceptive and exteroceptive cues (Small et al. 2008).

Interoceptive cues (gustatory, olfactory, auditory, and somatosensory cues) are those that come from inside the body and are directly involved in the flavor experience (Drew et al. 2023; Spence et al. 2010). These include dissolved tastants (five primary tastes), olfactory receptors (smell through the nose), and concomitant tactile stimulation (texture, pain, temperature) (Drew et al. 2023). Additionally, tactile sensations in the mouth cause a phenomenon known as “oral capture,” where the brain processes taste, smell, and texture signals as if they are coming from the entire mouth (Small et al. 2008). This makes the flavor feel distributed across the whole oral cavity, rather than being confined to specific areas, creating a unified flavor experience (Drew et al. 2023; Small et al. 2008).

It is important to note that taste and smell molecules are present in the mouth at the same time, which creates an illusion called “odor referral” (Fondberg et al. 2018). This means it is often mistakenly thought of smells to be actually tastes, confusing the signals from consumers’ sense of smell with those from their sense of taste (Fondberg et al. 2018).

The second category of multisensory contributions to flavor experience focuses on exteroceptive cues, sensory information from outside the body (Drew et al. 2023). These include visual cues like color and shape, orthonasal olfaction (smelling through the nose), and auditory cues like music in restaurants or sounds during food preparation (Drew et al. 2023; Spence et al. 2010). Such cues shape our expectations about flavor based on cross‐modal associations we have formed through repeated exposure to food from an early age (Schaal 2000).

Additionally, extrinsic product cues (such as labels, packaging, or context) also influence flavor by generating strong expectations, affecting how consumers perceive and enjoy food (Cardello 2007; Piqueras‐Fiszman and Spence 2015). These expectations can come from various sources (Deliza and MacFIE 1996). Visual aspects of a food product are essential in the selection and the acceptance of food products (Pereira and van der Bilt 2016).

Research on beer has shown that hop‐derived compounds like linalool and sesquiterpenoids have an impact on the sensory profile of beer, affecting not only aroma but also its taste and mouthfeel (van Opstaele et al. 2010). Another study on beer revealed a direct connection between hop aroma and perceived bitterness, as well as mouthfeel (Oladokun et al. 2017). It was found that hop aroma significantly affects both the intensity and character of perceived bitterness, often enhancing attributes like harshness, astringency, and lingering bitterness (Oladokun et al. 2017). This interplay between taste and aroma also extends to mouthfeel, with certain hop aromas contributing to sensations such as tingling and mouth‐coating effects, which impact the overall sensory experience of the beer (Oladokun et al. 2017).

Further exploring the role of sensory cues, research on visual cues influencing taste perception using mixed‐reality technology revealed that specific colors, shapes, and animations are associated with certain tastes (Huisman et al. 2016). For example, sweetness is linked to red and rounded shapes, whereas sourness is linked to green, angular shapes, and fast animations (Huisman et al. 2016). Visual cues can strongly influence how we experience food and what we expect from it. This has been confirmed through crowdsourcing and lab studies. This innovative approach suggests that mixed reality can enhance flavor perception, offering new possibilities in culinary presentation and product design (Huisman et al. 2016).

Research focusing on the labeling and sensory anticipation showed that when products are labeled as traditional or non‐traditional, these labels influence consumers’ expectations and, consequently, their sensory experiences (Siret and Issanchou 2000). In a study of “pâté de campagne,” it was observed that labels had a more pronounced impact on expectation scores based on visual examination than on actual liking scores after tasting (Siret and Issanchou 2000). Consumers’ expectations, influenced by these labels, can influence their perception. For example, products marketed as traditional often led consumers to expect higher quality, resulting in more positive sensory evaluations. However, if the actual taste and texture did not meet these elevated expectations, mainly when the product was worse than expected, consumer satisfaction was more negatively affected (Siret and Issanchou 2000).

Sensory expectation is key in shaping how we perceive the texture of food and beverages, which, in turn, affects our overall sensory experience (Labbe et al. 2006). The study revealed how the sense of smell affects the perception of the flavor of familiar bitter cocoa beverages and unfamiliar bitter milk (Labbe et al. 2006). The addition of cocoa aroma made the bitter cocoa drinks taste even more bitter, whereas the vanilla aroma triggered a sweeter perception in individuals. The study also found that people who are hesitant to try new things (neophobic) perceived bitterness more strongly when vanilla flavoring was added to bitter milk. Another study examined the impact of cocoa and vanilla aromas on beverage flavors (Labbe et al. 2006). Depending on the added aroma, for example, vanilla or cocoa, the drinks can taste sweeter or more bitter. The vanilla scent enhances the perception of sweetness, whereas the cocoa aroma gives the drinks a more bitter flavor (Labbe et al. 2006). This suggests that specific aromas can enhance particular taste qualities. However, when panelists evaluated the drinks while wearing a nose clip, there were no differences in perceived sweetness and bitterness. This suggests that although aroma is crucial for perceiving sweetness and bitterness, it does not have a substantial impact on the mouthfeel of the beverage in the absence of olfactory stimuli (Labbe et al. 2006). These findings emphasize how important aroma is in shaping the human experience of food and drinks, especially in enhancing or adjusting specific taste qualities (Labbe et al. 2006).

## 5 Mouthfeel Assessment

The traditional methods of mouthfeel assessment usually rely on sensory evaluation (sensory panels), utilizing a range of well‐established methods that offer valuable insights into human food perception (Ditschun et al. 2025; Fox et al. 2022). The most commonly used sensory tests include (a) discrimination tests, which assess overall differences between products (Ditschun et al. 2025; Langstaff et al. 1991a); (b) descriptive tests, which characterize specific sensory attributes; and (c) hedonic or affective tests, which measure consumer acceptance and satisfaction (H. Lawless and Heymann 2010) and temporal profiling (Cliff and Noble 1990; Ditschun et al. 2025). However, trained sensory panels are expensive and time‐consuming (Fox et al. 2021).

Instrumental techniques, such as **rheology** (which measures flow characteristics), have been successfully correlated with certain mouthfeel attributes like thickness, mouth coating, and stickiness. However, they fall short when more complex attributes are considered (Fox et al. 2021; Prakash et al. 2013; Selway and Stokes 2014).

Another method to predict the sensory properties of food and beverages is **soft tribology**, which studies friction, lubrication, and wear on soft surfaces (Shewan et al. 2020). It refers to the measurement of friction between two deformable or compliant surfaces, with the friction being evaluated as a function of speed (Fox et al. 2021). This approach helps assess how food behaves in the mouth, particularly in terms of texture and overall mouthfeel. Although there are known links between tribological data and sensory perception, they are often specific to certain formulations and setups, limiting broader application (Shewan et al. 2020). Although the method of soft tribology can be effectively used to evaluate the sensory properties of semi‐fluid food products like custards, yogurts, and thickened creams (Selway and Stokes 2013). Future research should refine formulation designs, integrate with techniques like rheology, and include saliva in studies to better simulate real conditions and improve sensory predictions (Fox et al. 2021; Shewan et al. 2020).

**E‐tongues** (ETs) have been used to collect data on sensory attributes like sourness, bitterness, and astringency in products such as beers, wines, and teas (Kaneda et al. 2003; Polshin et al. 2010; Rudnitskaya et al. 2009, Rudnitskaya et al. 2010). Both e‐noses and ETs simulate human taste and smell sensors (gas and liquid sensors), as well as their interaction with the brain, to analyze complex flavors (Baldwin et al. 2011). For example, research on Madeira wines demonstrates the effective use of an ET in combination with HPLC to evaluate sensory properties, particularly in detecting organic acids and phenolic compounds (Rudnitskaya et al. 2010). The ET system accurately predicted wine age (with a precision of 1.8 years) and successfully quantified tartaric, citric, and vanillic acids, proving its value as a fast and cost‐effective tool for wine quality control (Rudnitskaya et al. 2010). Similarly, ETs have been shown to accurately assess and predict sensory characteristics of beer, including bitterness, sweetness, sourness, and body (Rudnitskaya et al. 2009). A study involving 50 Belgian and Dutch beers included dark and lager beers, ales, white (wheat), lambic fruit, and Trappist beers; however, the alcohol content was not specified for each beer. It demonstrated the application of an ET with potentiometric chemical sensors for the precise analysis of key physicochemical parameters such as bitterness, alcohol content, polyphenol levels, and real extract (Polshin et al. 2010). The ET exhibited strong sensitivity to isomerized hop extracts, making it a valuable tool for quickly and reliably assessing beer quality, especially in predicting bitterness and other sensory parameters (Polshin et al. 2010). In the context of fruit‐based products, research on apple juice confirmed that e‐nose and ET sensors can effectively evaluate sensory properties by correlating sensory panel data with consumer preferences (Baldwin et al. 2011). ETs provide a precise prediction of consumer acceptance, allowing for better control of key sensory attributes (Baldwin et al. 2011).

Another analytical technique for flavor evaluation is QCM technology. Kaneda et al. (2003) developed two methods for measuring astringency in beverages using lipid‐coated QCM (tannins) and gelatin‐immobilized QCM (polyphenols). The sensors effectively detected astringent tannins and polyphenols in red wines, Japanese green teas, and beers, with results closely aligning with sensory evaluations (Kaneda et al. 2003). Another research showed the lipid‐coated QCM can be used to measure the body, bitterness, smoothness, and astringency of the beer (Kaneda et al. 2002, 2005).

## 6 Mouthfeel in Beer

Mouthfeel plays a crucial role in product acceptance across all food and beverage categories. In this article, beer is used as a showcase example, particularly in light of the growing non‐ and low‐alcoholic beverage segment, to demonstrate how mouthfeel influences sensory perception and consumer satisfaction.

Beer is the oldest alcoholic beverage (Buiatti 2009). The practice of making beer has been utilized for thousands of years across various cultures and remains significant both in popularity and economic impact (Gil et al. 2022; Koller and Perkins 2022). Beer is consistently the most‐consumed alcoholic beverage in the world in terms of volume (Betancur et al. 2020). It is the third most popular drink globally, after water and tea (Agorastos, Klosse, et al. 2023; Piazzon et al. 2010). In 2023, the global beer production amounted to about 1.88 billion hectoliters (Statista 2025). The market share of craft brewers is still increasing (Fox et al. 2022), and microbrew beers are getting more popular (Gabrielyan et al. 2014). The rise of the craft beer movement around the world has changed the traditional patterns of beer (Betancur et al. 2020).

The core attributes of the beverage have become more critical in consumers’ purchasing decisions (Gabrielyan et al. 2014). The hoppiness and overall beer flavor have a critical and positive impact on willingness to pay (Gabrielyan et al. 2014). Additionally, the last two decades have seen several gradual but steady changes in consumers’ drinking patterns (Betancur et al. 2020). Although beer is one of the most popular beverages, its global consumption has been decreasing worldwide in recent years, though the consumption of non‐alcoholic beers is increasing (Krebs et al. 2019). Consumers’ health and wellness awareness has increased significantly over the last half‐decade (Krebs et al. 2019), influencing their choices. This segment covers non‐alcoholic (NA) and low‐alcohol (LA) beer, and it has grown significantly in the past years, with further growth being (Bellut and Arendt 2019; Fox et al. 2022).

It is due to changing consumer habits, and non‐alcoholic beer is the fastest‐growing market within the beverage industry (Bellut and Arendt 2019; Krebs et al. 2019). However, the de‐alcoholized beer shows organoleptic challenges and lacks acceptance from many consumers (Bellut and Arendt 2019; Fox et al. 2022; Krebs et al. 2019). Consumers often describe the sensory characteristics of non‐alcoholic beers, particularly palate fullness, mouthfeel, and the balance between sweetness and sourness, as unbalanced and lacking (Bellut and Arendt 2019; Fox et al. 2022; Krebs et al. 2019).

Beer is a compound beverage with a general composition of water, carbon dioxide, alcohol, and extracts (low molar mass compounds and several polymers) (Agorastos, Klosse, et al. 2023; Fox et al. 2022; Krebs et al. 2021, 2019). Carbohydrates, ethanol, polyphenols, carbon dioxide, and glycerol are present in more significant quantities in beer (Agorastos, Klosse, et al. 2023), in addition to about 800 organic compounds present (Betancur et al. 2020). A large proportion of constituents in beer are either present in raw materials (water, malt, hops) or are byproducts of yeast metabolism during fermentation (Betancur et al. 2020; Buiatti 2009). However, the specific contributions of various beer components to the sensations of mouthfeel remain mostly unexplored (Agorastos, Klosse, et al. 2023).

Mouthfeel is a significant parameter for the total perception of beer flavor and is essential in the overall beer‐drinking experience (Agorastos, Klosse, et al. 2023). Palate fullness, body, and mouthfeel are sensory descriptors widely used by scientists to describe beer (Fox et al. 2022; Ivanova et al. 2022, 2023; Krebs et al. 2021, 2019). It encompasses various sensory attributes such as carbonation, astringency, smoothness, fullness, and mouthcoating (Agorastos, Klosse, et al. 2023; Krebs et al. 2019). The knowledge of how compounds influence the mouthfeel of beer can help brewers design beverage products that are more appealing to consumers (Agorastos, Klosse, et al. 2023). On the basis of Klosse's model, we can classify mouthfeel into three dimensions: coating, drying, and contracting (Agorastos, Klosse, et al. 2023; P. Klosse 2013). This model can be used to predict mouthfeel sensations building up on the chemical composition and other characteristics of beers (Agorastos, Klosse, et al. 2023). Additionally, range of methods can be applied to evaluate the mouthfeel of beer. These include traditional approaches such as sensory evaluation (Fox et al. 2021) and instrumental techniques like soft tribology (Fox et al. 2021), ETs (Rudnitskaya et al. 2009), and QCM (Kaneda et al. 2002, 2003, 2005).

### 6.1 Key Compounds Impacting Mouthfeel in Beer

Mouthfeel is associated with the sensory attributes of carbonation, astringency, drying, smoothness, fullness, watery, warming, and mouth‐coating (Fox et al. 2022; Guinard and Mazzucchelli 1996; Krebs et al. 2019). Different substance classes have been found to affect the mouthfeel, palate fullness, and body of beer (Ivanova et al. 2022, 2023), and these substances are generally classified according to their molar mass (Krebs et al. 2019). This applies to LMW and high molecular weight (HMW) groups.

The LMW components of beer include ethanol, glycerol, carbon dioxide (CO_2_) (Krebs et al. 2019; Langstaff and Lewis 1993), organic acids, esters, higher alcohols, aldehydes (Baert et al. 2012), and ketones (diacetyl). A range of macromolecular compounds (HMW) includes proteins and peptides, polysaccharides (dextrins, maltodextrin, and β‐glucans), glycoproteins, tannins (polyphenols) (Krebs et al. 2019), and lipids. They influence the palate fullness of beer (Krebs et al. 2019). Additionally, the water and minerals in beer affect mouthfeel (Eßlinger and Narziß 2009; Parker 2012).

#### 6.1.1 LMW Compounds

Ethanol, the main byproduct of yeast metabolism, plays a significant role in enhancing the body of beer (Ivanova et al. 2023; Langstaff and Lewis 1993). It increases the viscosity, contributing to a fuller or thicker mouthfeel, and is a critical factor in the beer's overall texture. Additionally, ethanol produces a warming, full‐bodied, and lingering sensations when consumed (Bamforth 2023; Liu et al. 2024). Its influence extends beyond mouthfeel, as it greatly impacts flavor release and sensory perception in alcoholic beverages. Studies have demonstrated that ethanol can have a strong impact on flavor release and chemosensory perception (Ickes and Cadwallader 2017). The presence of ethanol enhanced the release of aroma compounds, mainly aldehydes and esters, into the headspace and increased viscosity (Liu et al. 2024). Variations in ethanol levels can alter how consumers perceive aroma, taste, and mouthfeel in alcoholic beverages (Ickes and Cadwallader 2017). Additionally, some research has demonstrated that ethanol positively contributes to the perception of sweetness in beer.

Glycerol (propane‐1,2,3‐triol), a byproduct of yeast fermentation (S. Q. Liu 2015), is recognized for its naturally sweet taste (S. Q. Liu 2015) and viscosity, and in high amounts can influence the taste and mouthfeel of beer (S. Q. Liu 2015). In beverages, glycerol enhances smoothness and contributes to a fuller, richer mouthfeel while enhancing flavor intensity (Ramsey 2020; Zhao et al. 2015). This is particularly important in non‐alcoholic or low‐alcohol beers (Ramsey 2020; Zhao et al. 2015), where glycerol can replace some of the sensory qualities typically provided by ethanol (Perpète and Collin 2000; Ramsey 2020). Studies on Riesling wines indicated that glycerol can contribute to the perception of a fuller and more viscous texture, although the effect varies depending on the wine's composition (Gawel et al. 2007). Notably, the influence of glycerol on the mouthfeel, particularly in terms of body and viscosity, is less predictable compared to ethanol (Gawel et al. 2007). Further research on wine highlights that glycerol significantly enhances its mouthfeel by improving smoothness, body, and viscosity, which is crucial when ethanol content is reduced (Tilloy et al. 2014). These effects help maintain a balanced and high‐quality sensory experience (Tilloy et al. 2014). It is important to note that although glycerol positively affects the mouthfeel of wine, its impact on beer may differ due to the distinct composition and interactions of beer constituents.

Carbonation plays a significant role in influencing the mouthfeel of beer (Ivanova et al. 2023). The level of carbonation, the concentration of CO_2_, contributes to several aspects like tactile sensation, such as sting, bubble size, and foam volume (Ivanova et al. 2023); additionally, it can enhance flavors like sourness and saltiness while diminishing others (Vlădescu et al. 2021). Nevertheless, an excessive or insufficient level of carbonation may lead to a general flavor imbalance (Oddone 2022; Vlădescu et al. 2021). Studies on mammals suggest that carbonation triggers both somatosensory and chemosensory responses and even activates taste neurons (Vlădescu et al. 2021). When discussing carbonation, it is important to address the concept of “carbonation bite.” Although it is commonly believed that this sensation results from the physical stimulation of bubbles in the mouth, research has shown that carbonation bite primarily arises from the formation of carbonic acid in the oral mucosa (Wise et al. 2013).

Organic acids are commonly present in beer and are critical for the taste (Agorastos, Klosse, et al. 2023). Organic acids are key components in alcoholic beverages and significantly impact their sensory qualities, including flavor and mouthfeel (Yan et al. 2024). In beer production, organic acids are essential as they influence the flavor, color, and aroma while also affecting the pH level, together with carbonic acid (Agorastos, Klosse, et al. 2023), which directly impacts the beer's overall quality (Rodrigues et al. 2010; Yan et al. 2024). The optimal concentration of organic acids in beer contributes to a refreshing crispness, enhancing its sensory appeal (Dysvik et al. 2020; Yan et al. 2024). In contrast, insufficient levels can lead to an undesirable, overly viscous mouthfeel (Dysvik et al. 2020; Yan et al. 2024). Conversely, excessive organic acids result in an unbalanced taste profile, lacking the smoothness and harmony (Dysvik et al. 2020; Yan et al. 2024). Organic acids are classified into volatile and non‐volatile groups. The volatile acids include acetic acid, butanoic acid, phenylacetic acid, 2‐methylpropionic acid, 3‐methylbutanoic acid, and geranic acid (Féchir et al. 2021; Yan et al. 2024). These acids are notable for imparting sour aromas (Féchir et al. 2021; Yan et al. 2024). Acetic acid contributes a pungent sourness and sharp mouthfeel, whereas butanoic acid adds a rancid, cheesy note (Féchir et al. 2021; Yan et al. 2024). Geranic acid, derived from hops and primarily found in the Sorachi Ace variety, does exhibit subdued odor‐active properties; however, it serves as an aromatic enhancer for hop‐derived terpenoids, such as linalool and geraniol (Ohashi et al. 2021; Sanekata et al. 2018; Yan et al. 2024). Non‐volatile acids, such as malic acid, lactic acid, succinic acid, ascorbic acid, citric acid, and iso‐alpha acid, impart sour, bitter, and umami characteristics (Rodrigues et al. 2010; Yan et al. 2024). Succinic acid, produced by *Saccharomyces cerevisiae*, enhances the umami flavor at low threshold concentrations (700 µmol/kg), but at higher levels (900 µmol/kg), it contributes to sour taste (Rotzoll et al. 2006; Yan et al. 2024). Iso‐alpha acids are important acidic compounds in beer that contribute to its bitterness. Alpha acids, found in hops, are the precursors of iso‐alpha acids formed during brewing. The *cis* form of iso‐alpha acids is more bitter than the *trans* form (Bossaert et al. 2022; Yan et al. 2024). Additionally, it is worth mentioning the level of iso‐alpha acids closely correlates with the bitterness of beer and is quantified using the International Bitterness Units (IBUs) (Luo et al. 2020).

Esters are the most aroma‐active volatile compounds in beer, and they have a positive impact on the overall beer flavor (S. Q. Liu 2015). They are the vital aroma compounds produced by yeast during primary fermentation through enzymatic chemical condensation of organic acids and alcohols (Pires et al. 2014). Esters deliver fruity aromas in beer; however, excessive levels of esters can lead to overly fruity, fermented off‐flavor (S. Q. Liu 2015), and among the dozens of different esters that can be found, six are considered the most important aromatic contributors: ethyl acetate (solvent‐like smell), isoamyl acetate (banana‐like smell), isobutyl acetate (fruity smell), phenyl ethyl acetate (rose‐ and honey‐like smell), ethyl hexanoate (sweet apple smell), and ethyl octanoate (sour apple smell) (Pires et al. 2014). These compounds significantly contribute to the overall sensory perception of beer, especially its fruity character (Pires et al. 2014). Although there is extensive research and literature available on esters and their aromatic properties, there is limited information about their influence on mouthfeel. However, their impact on mouthfeel becomes more evident when combined with ethanol, highlighting the interaction between esters and alcohol in shaping the sensory experience (Liu et al. 2024). Esters, along with ethanol, are key to improving the sensory experience of alcohol‐free beers (Liu et al. 2024). Although the research focused on ethanol's influence on mouthfeel, it also highlights that esters, in the presence of ethanol, play a role in enhancing this effect (Liu et al. 2024).

Higher alcohols—the type of higher alcohols formed is determined by the type of amino acids present (S. Q. Liu 2015). The most common alcohol‐containing amino acids are threonine (*n*‐propanol), valine (isobutanol), leucine (isoamyl alcohol), isoleucine (active amyl alcohol), and phenylalanine (2‐phenyl ethyl alcohol) (S. Q. Liu 2015). Unfortunately, there is limited research about the influence of higher alcohol on the mouthfeel of beer.

Ketones are present in beer but generally found in very low concentrations (S. Q. Liu 2015). This includes 2‐nonanone, β‐damascenone, β‐ionone, 2,3‐butanedione (diacetyl), and 2,3‐pentanedione (Martins et al. 2017), the vicinal diketones diacetyl and 2,3‐pentanedione. Diacetyl (2,3‐butanedione) is formed during fermentation as a byproduct (Krogerus and Gibson 2013; Parker 2012), and it is a very potent odor‐active compound (S. Q. Liu 2015) and is considered an off‐flavor (Bitew and Andualem 2024). It is known for its butter‐ or butterscotch‐toffee‐like flavor (Krogerus and Gibson 2013; Parker 2012), and so it is associated with a thicker sensation on the tongue (Hughes 2009).

Aldehydes such as acetaldehyde, hexanal, (*E*)‐2‐nonenal, furfural, 2‐methylpropanal (isobutanal), 2‐methylbutanal (amyl aldehyde), 3‐methylbutanal (isoamyl aldehyde), 3‐methylthiopropanal (methional), 2‐phenylacetaldehyde, and benzaldehyde are also present in beer (S. Q. Liu 2015). These aldehydes affect beer flavor by imparting organoleptic notes (S. Q. Liu 2015). They play a significant role in the sensory perception of beer aging (Baert et al. 2012) as they contribute to flavor changes, such as bitterness, harshness, sweetness, or caramel‐like notes, as well as to aroma degradation during the staling process (Baert et al. 2012).

Water chemistry—Water is the primary ingredient and foundation for a successful brewing process (Eßlinger and Narziß 2009). The soft water in Pilsen, with low levels of minerals except for carbonates (CO_3_^2−^), helps create lagers with a smooth, rounded mouthfeel (Parker 2012). Nowadays, many breweries adjust the mineral content of water to match the high mineral levels of Burton‐on‐Trent water (Burtonization process) for certain beer styles, or they remove salts to mimic Pilsen‐style water (Parker 2012). Some ions directly affect the brewing process, whereas others impact the beer's flavor and mouthfeel (Parker 2012). Calcium (Ca^2+^) helps, among others, control wort pH and supports enzyme activity, but it has little influence on flavor (Parker 2012). Chloride (Cl^−^) and sulfate (SO_4_^2−^) ions, however, are essential for the taste and mouthfeel of beer, with many brewers carefully balancing the chloride‐to‐sulfate ratio in brewing water. Sulfates enhance the dry, crisp mouthfeel, whereas chlorides provide body and fullness (Parker 2012). Despite this, there is limited research on the exact effects of these minerals on beer's mouthfeel (Parker 2012).

In the spontaneous fermentation of acidic beers like lambics, non‐*Saccharomyces* yeasts, particularly *Brettanomyces*, can ferment malto‐oligosaccharides sugars that regular brewing yeast (*S. cerevisiae*) cannot metabolize and hence leaves behind (S. Q. Liu 2015; Verachtert and Derdelinckx 2014). This can thin the beer's body, potentially affecting the mouthfeel negatively (Baert et al. 2012; S. Q. Liu 2015).

Additionally, during yeast autolysis in fermentation (both spontaneous and controlled), amino acids (e.g., glutamic acid), nucleotides (e.g., guanosine monophosphate and inosine monophosphate), and glutathione (GSH) are formed (S. Q. Liu 2015). It has been reported that GSH delivers kokumi flavor (S. Q. Liu 2015), contributing to a more complex and satisfying flavor profile (Mizuta et al. 2021), which is enhanced by glutamic acid and inosine monophosphate (S. Q. Liu 2015).

#### 6.1.2 HMW Compounds

The **proteins and polypeptides** present in beer serve a multitude of functions. Proteins are large, complex molecules build of amino acids linked via peptide bonds (Keskin et al. 2008) that affect many properties of a beer, including color, flavor, and stability. In addition, proteins are one of the key contributors to the mouthfeel of beer (Fox et al. 2022). These compounds are also catabolized by yeast for energy, reproduction, and enzyme generation (Koller and Perkins 2022). HMW (10–20 kDa) polypeptides contribute to the beer flavor by increasing smoothness and softness together with decreasing astringency (Kato et al. 2021). LMW polypeptides (2–3 kDa) contributed to the overall body and were associated with boosting umami flavors and enhancing mouthfeel attributes like “roundness” and viscosity (Kato et al. 2021).

**Polysaccharides**, such as beta‐glucans, dextrins, and maltodextrins, play essential roles in shaping the mouthfeel, viscosity, and body of beer, each contributing differently to the sensory experience. **Beta‐glucans** are known for their significant impact on beer viscosity and perceived thickness, enhancing the body and mouthfeel of the beverage (Bettenhausen et al. 2018). High levels of beta‐glucans can result in a creamier, fuller mouthfeel, which is often desirable in certain beer styles (Bettenhausen et al. 2018). Experimental studies have shown that spiking beer with beta‐glucans increases its viscosity and creates a thicker mouthfeel (Krebs et al. 2019). However, excessive beta‐glucan levels can lead to an overly “slimy” or viscous texture, which may negatively affect sensory perception (Moreno Ravelo et al. 2024). When beta‐glucan concentrations are kept within moderate ranges (100–350 mg/L) and balanced with other polysaccharides such as dextrins, they enhance the palate fullness and contribute positively to mouthfeel quality (Moreno Ravelo et al. 2024). **Dextrins**, non‐fermentable polysaccharides, are key contributors to beer's body and fullness (Fox et al. 2022; Moreno Ravelo et al. 2024). They increase viscosity and enhance the pleasant sensations of palate fullness without being metabolized during fermentation (Moreno Ravelo et al. 2024). Research highlights their importance in creating a desirable mouthfeel (Fox et al. 2022). However, an imbalance, such as low dextrin levels combined with high beta‐glucan concentrations, can diminish the quality of palate fullness (Moreno Ravelo et al. 2024). **Maltodextrins**, hydrolyzed starch, are another important polysaccharide (Muhamad et al. 2018). They play a crucial role in enhancing palate fullness and improving key mouthfeel attributes. Studies have shown that maltodextrins with a degree of polymerization (DP) ranging from 2 to 10 contribute to a smooth, lasting, and pleasant mouthfeel (Kato et al. 2021). Their addition also decreases the sensation of astringency, resulting in an overall more balanced and enjoyable sensory experience (Muhamad et al. 2018). In low‐malt beer, the impact of maltodextrins is further amplified when combined with HMW and LMW polypeptide fractions (Kato et al. 2021). Together, these components significantly improve softness and smoothness while reducing undesirable attributes like roughness and astringency, creating a more harmonious and richer mouthfeel (Kato et al. 2021). This synergy highlights the importance of maltodextrins, both individually and in combination with polypeptides, in shaping the sensory profile of beer.

The perception of fat is a complex process that shapes the sensory experience of beverages and plays a key role in the taste, texture, nutrition, and aroma of food products (Drewnowski and Almiron‐Roig 2010; Vossen et al. 2024). And as much as **lipids** do influence a creamy mouthfeel by contributing to the perceived richness and fullness (Bettenhausen et al. 2018), there are limited resources focusing on the presence of lipids in beers and their influence on mouthfeel.

**Polyphenols** are one of the key contributors to the beer's mouthfeel (Fox et al. 2022; Goiris et al. 2014). Beer contains an appreciable number of phenolic compounds, originating mainly from barley (about 70%) and hop (about 30%) (Callemien et al. 2005; Goupy et al. 1999; Piazzon et al. 2010). Beer rich in phenolic antioxidants shows higher quality, more stable sensory properties (flavor, aroma), foam stability, and longer shelf life with respect to beer with lower antioxidant activity (Callemien et al. 2005; Goupy et al. 1999; Piazzon et al. 2010). The addition of polyphenols to beer induced harsh bitterness and increased astringency (Habschied et al. 2021). Polyphenol content along with antioxidant activity increased in the following order: dealcoholized < lager < pilsner < wheat < ale < abbey < bock (Callemien et al. 2005; Goupy et al. 1999; Piazzon et al. 2010). Both polyphenol content and antioxidant activity have been reported to be lower in alcohol‐free beers with respect to strong and dark beers (Gorjanović et al. 2010; Piazzon et al. 2010). Tannins (class of polyphenols) provide astringent sensations, a complex sensory profile, characterized by drying, roughing, and puckering (Habschied et al. 2021; Siebert and Chassy 2004). An addition of flavonol glycosides and prenylated hop flavonoids to beer influenced the mouthfeel and improved flavor stability (Schoenberger and Haas Group 2006). Rutin, which delivers a velvet‐like astringency, showed an overwhelmingly high taste activity due to its considerably low taste threshold (Li et al. 2019).

### 6.2 The Impact of Hop‐Derived Compounds on the Flavor of Beer

Hop (*Humulus lupulus* L.) is a climbing herbaceous perennial vine that belongs to the family Cannabaceae (Habschied et al. 2021). Only female plants (pine‐like cones) are used for brewing purposes (Almaguer et al. 2014; Habschied et al. 2021). In the brewing world, hops are mainly appreciated for the content and composition of the bitter acids, alpha acids (humulones) (De Keukeleire 2000) and beta‐acids (or *lupulone*) (De Keukeleire 2000; Habschied et al. 2021), and the essential oils that are produced and accumulated in the lupulin glands of the hop cones (Calvert et al. 2024; Van Holle et al. 2021). Bitterness is one of the primary aspects of flavor contributed to beer by hops (Calvert et al. 2024; Schönberger and Kostelecky 2011). Research in brewing revealed that iso‐alpha acids, a product of chemical conversion during the brewing process, the thermal isomerization of the alpha acids (De Keukeleire 2000), are the main contributor to beer bitterness, but they are not the only source of bitterness in beer (Schönberger and Kostelecky 2011). Furthermore, hops exhibit preservative properties on account of their beta‐acids, which have antimicrobial properties, especially against gram‐positive bacteria (Schönberger and Kostelecky 2011; Shen and Sofos 2008). Although they were never considered to contribute to the flavor, some beta‐acid transformation products formed during the wort boil might contribute to the bitterness as well (Haseleu et al. 2009; Schönberger and Kostelecky 2011). This includes cohulupone, two tricyclocolupone epimers, two dehydrotricyclocolupone epimers, hulupinic acid, two hydroxytricyclocolupone epimers, and two hydroperoxytricyclocolupone epimers (Haseleu et al. 2009; Schönberger and Kostelecky 2011). Another group of compounds that can contribute to bitterness and astringency, depending on their DP and concentration in the beer, are hop polyphenols (Schönberger and Kostelecky 2011). Hop polyphenols interact with high molecular proteins of wort, forming complexes that fall into the precipitate and thus improve the clarification of wort and beer (Bober et al. 2020; Goiris et al. 2014).

The key polyphenols commonly found in hops are xanthohumol, isoxanthohumol, rutin, quercetin, kaempferol, catechin, epicatechin, proanthocyanidins, tannins, ferulic acid, caffeic acid, *p*‐coumaric acid, gallic acid, chlorogenic acid, and vanillic acid (De Keukeleire 2000; Magalhães et al. 2008; Oladokun et al. 2017). These polyphenols influence beer's sensory properties, including bitterness, astringency, and mouthfeel, and contribute to its antioxidant capacity. Astringency is a tactile sensation characterized by puckering, tightening, and dryness in the mouth, commonly caused by polyphenols (Guerreiro et al. 2022). Although recognized as a trigeminal sensation, the precise molecular mechanisms behind it remain unclear (Guerreiro et al. 2022). Additionally, it is uncertain whether the various sub‐qualities of astringency, such as velvet, silky, adhesive, puckering, harshness, and dryness, are perceived through different mechanisms (Guerreiro et al. 2022). Research on the addition of xanthohumol to pale ale and dark beers showed that it contributes to a smoother bitterness and improves the overall mouthfeel and flavor stability of beer (Magalhães et al. 2008). Astringent compounds include the phenolic compounds, in particular, tannins (Guerreiro et al. 2022). Both gallotannins and ellagitannins have been reported to elicit astringency (Guerreiro et al. 2022). The investigation into the interaction between human saliva and astringent compounds has shown that the effect of saliva on epicatechin (EC) and rutin is minimal (Rossetti et al. 2008). Although rutin does play a role in contributing to astringency, its quantitative impact is comparatively minor when assessed alongside other polyphenols (Rossetti et al. 2008). In addition to tannins, numerous studies confirmed that astringency could also be elicited by flavonols (Guerreiro et al. 2022).

Hops contain essential oils (0.5%–4% v/w) (Lafontaine and Shellhammer 2019), which are volatile compounds that play a crucial role in shaping the aroma and flavor of beer (De Keukeleire 2000). However, certain hop varieties, particularly those originating from the United States, are known for their high oil content, for example, Zeus variety (up to 4.5% v/w) (BarthHaas 2025), and some even reaching 5.1% (Haunold et al. 1983). The three main classes of compounds in hop essential oil are hydrocarbons, oxygenated compounds, and sulfur‐containing compounds (De Keukeleire 2000; Duarte et al. 2023). Hydrocarbons typically constitute 50%–80% of the total oil content, with monoterpenes such as myrcene and sesquiterpenes like α‐humulene and β‐caryophyllene being the most prevalent constituents. Oxygenated compounds, including terpene alcohols like linalool and geraniol, contribute significantly to hop aroma and flavor (De Keukeleire 2000; Duarte et al. 2023). Although sulfur‐containing compounds are present in much smaller amounts (up to 1% of the total oil), they have a substantial impact on aroma due to their low perception thresholds (De Keukeleire 2000; Duarte et al. 2023). The perception of hop volatiles varies depending on their concentrations, combinations, and threshold levels in different beer matrices (Dietz et al. 2020, 2019; Mikyška et al. 2024). This variability can lead to synergistic or antagonistic effects among individual odorants, contributing to the overall sensory experience (Dietz et al. 2020, 2019; Mikyška et al. 2024). Research has shown that the presence of hop aromas can alter taste and mouthfeel, contributing to a complete sensory experience of hop flavor (Dietz et al. 2020). Linalool has also been found to be involved in aroma–taste interactions, modifying the perception of bitterness qualities in beer (Dietz et al. 2020). Additionally, oxygenated sesquiterpenoids are suggested to be accountable for a tingling and irritating sensation indicating the activation of trigeminal receptors (Dietz et al. 2020). Despite the extensive research on hops and brewing processes, there is a limited number of studies examining hop and hop‐derived compounds and their influence on mouthfeel.

## 7 Conclusions

Mouthfeel is a complex and multidimensional sensation that still lacks a clear and universally accepted definition. It involves a combination of physical and chemical interactions in the mouth, making it difficult to isolate or measure using a single sensory or instrumental method. It includes tactile, thermal, and chemesthetic sensations and is shaped by both the somatosensory and chemosensory systems. Mouthfeel covers key sensory attributes such as smoothness, crunchiness, astringency, and warming and cooling sensations. These perceptions result from interactions among food or beverage components, oral receptors, and saliva, making mouthfeel inherently multisensory and highly context‐dependent. Unlike taste and aroma, mouthfeel lacks standardized measurement techniques and remains more difficult to quantify, despite its significant impact on consumer preference, sensory satisfaction, and overall product acceptance.

The authors propose a visual structure (Figure 1c) to support a better understanding of mouthfeel as a sensory experience resulting from both non‐volatile and volatile compounds, detected through the chemosensory and somatosensory systems. Signals from the oral chemosensory system, particularly via the trigeminal nerve, contribute to sensations such as cooling, burning, drying, and warming. At the same time, the oral somatosensory system processes

Physical sensations, including:

– Proprioception and kinesthesis, which relate to the movement and position of food in the mouth and help describe attributes, such as elasticity, sharpness, crunchiness, and structure‐related sound (e.g., crispy textures) and

Tactile sensations, including:

Texture (creamy, thick, oily, smooth, gritty, and crunchy),

Pressure (soft, firm, and chewy), and

Temperature (hot and cold).

This proposed framework, presented in Figure 1c, is intended as a suggested structure based strictly on information available in the current literature.

Beer case study included in the article shows why mouthfeel is an important part of the sensory experience. To meet consumer expectations and follow current trends and market growth, mouthfeel is now seen as an essential sensory attribute in product development. This is especially true for non‐ and low‐alcoholic beers, where removing or reducing alcohol makes it harder to create the full‐bodied and balanced sensations typical of traditional beers. These products are often described as lacking body, leading to a watery and less satisfying mouthfeel.

Although recent developments in sensory science and instrumental methods like soft tribology and rheology support improved product formulation, there is still a need for further improvement and research. In particular, more precise and standardized evaluation methods are lacking, which limits the ability to fully understand and measure mouthfeel. This is crucial for enhancing product quality, consumer acceptance, and expectations.

## Author Contributions

**Katarzyna Wolinska‐Kennard**: writing – original draft, visualization, investigation, project administration. **Christina Schönberger**: supervision, writing – review and editing, conceptualization. **Adam Fenton**: resources, funding acquisition. **Aylin W. Sahin**: conceptualization, writing – review and editing, supervision, project administration.

## Conflicts of Interest

This work is part of the PhD thesis of Katarzyna Wolinska‐Kennard who is employed by BarthHaas Group. The authors Christina Schönberger and Adam Fenton are employees of BarthHaas Group.
